# Vaccine Effectiveness against SARS-CoV-2 among Household Contacts during Omicron BA.2–Dominant Period, Japan

**DOI:** 10.3201/eid3007.230968

**Published:** 2024-07

**Authors:** Tsuyoshi Ogata, Hideo Tanaka, Akemi Kon, Noriko Sakaibori, Emiko Tanaka

**Affiliations:** Itako Public Health Center of Ibaraki Prefectural Government, Itako, Japan (T. Ogata, A. Kon, N. Sakaibori, E. Tanaka);; Public Health Center of Neyagawa City, Neyagawa, Osaka, Japan (H. Tanaka)

**Keywords:** COVID-19, Contact tracing, COVID-19 vaccines, respiratory diseases, incidence, risk factors, SARS-CoV-2, SARS-CoV-2 variants, vaccine efficacy, vaccine effectiveness, infectiousness, household contact, attack rate, Omicron, BA.2, coronavirus disease, zoonoses, viruses, coronavirus, Japan

## Abstract

We calculated attack rates for household contacts of COVID-19 patients during the SARS-CoV-2 Omicron BA.2–dominant period in Japan. Attack rates among household contacts without recent (<3 months) vaccination was lower for contacts of index patients with complete vaccination than for contacts of index patients without complete vaccination, demonstrating indirect vaccine effectiveness.

BA.2 is a subvariant of the SARS-CoV-2 Omicron variant. XBB.1.5 and XBB.1.16, recombinants of the BA.2.10.1 and BA.2.75 sublineages, were circulating SARS-CoV-2 variants of interest in July 2023 (*1*,*2*), but few estimates of vaccine effectiveness (VE) against infectiousness of those variants were available.

On April 20, 2022, the government of Japan required local public health centers (PHCs) to be notified of all COVID-19 patients and to implement contact tracing (*3*). By that date, the jurisdiction of the Itako PHC (population ≈265,000) in Ibaraki Prefecture had 13,555 confirmed COVID-19 patients (5.1% of the population) (*4*). Among patients with confirmed COVID-19, a total of 2.0% were hospitalized during April–May 2022. 

In our previous study of the Omicron BA.1 dominant period in Itako (*5*), the household contact attack rate (HCAR) was lower for household contacts of index patients who were completely vaccinated than for household contacts of other index patients. However, the vaccination status of household contacts was associated with the vaccination status of the associated index patient (*5*). Therefore, adjustment, such as stratification for confounding by the contacts’ vaccination status, was needed for adequate evaluation of VE against infectiousness. This study aimed to assess VE against the infectiousness of the SARS-CoV-2 Omicron BA.2 variant among household contacts.

## The Study

We obtained information on COVID-19 patients and household contacts reported in the jurisdiction of the Itako PHC in Ibaraki Prefecture, Japan, during April 21–30, 2022. The epidemiologic investigation and data collection procedure on COVID-19 index patients was almost the same as that described in our previous study (*5*). The study design was approved by the Ibaraki Prefecture Epidemiologic Research Joint Ethics Review Committee (protocol no. R3-10). We performed statistical analyses by using R version 4.4.1 (The R Foundation for Statistical Computing, https://www.r-project.org).

Genomic sequencing surveillance showed that 90% of 5,630 variants of concern sampled in Japan during April 18–May 1 were BA.2 (*6*). In Ibaraki, sequencing of 69 samples collected during April 23–May 13 demonstrated that 65 (94%) samples were BA.2 and 4 (6%) were BA.1.

Itako PHC undertook or requested SARS-CoV-2 testing of household contacts who had any symptoms during their 5-day observational and quarantine period of a COVID-19 patient (*5*,*7*,*8*). Asymptomatic household contacts were not tested. During the study period, the PHC jurisdiction identified 729 confirmed COVID-19 cases (*4*) (Figure). After excluding 112 index cases without household members, we identified 617 infected household members, and a total of 1,575 household members, including cases and contacts. We deemed patients with the earliest symptom onset date as index cases in the household, which comprised 388 patients. Of 1,187 household contacts of the 388 index patients, we included 952 who had available data and no history of previous infection in the study. Of the 235 excluded household contacts, we excluded 7 because of a history of infection before the study period. Of the 952 contacts, 395 were infected; the overall HCAR was 41.5%.

**Figure:**
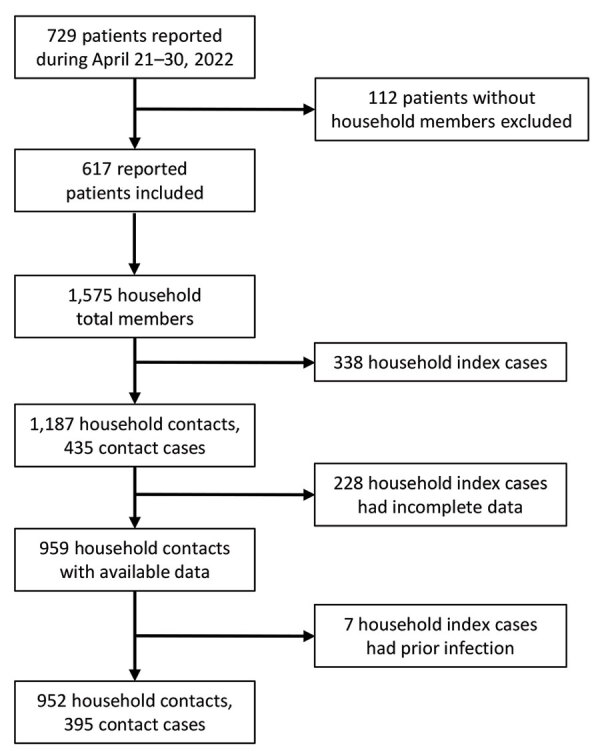
Flowchart of enrollment of index patients and household contacts in study of vaccine effectiveness against SARS-CoV-2 among household contacts during Omicron BA.2–dominant period, Japan.

Excluding health professionals, 82.5% of residents in Ibaraki had completed 2 COVID-19 vaccine doses, and 56.3% had received a booster dose by April 20, 2022 (*9*). Among initial vaccinations in Ibaraki, 81% were BNT162b2 vaccine (Pfizer-BioNTech, https://www.pfizer.com) and 19% mRNA-1273 vaccine (Moderna, https://www.modernatx.com) (*5*).

We stratified vaccination status on the basis of vaccination doses or recent vaccination, which we defined as receiving the last vaccine dose within 3 months. HCAR was 30.9% for contacts with 3-dose vaccination and 32.4% for contacts with recent vaccination, substantially lower than the 49.6% for contacts without complete vaccination (0–1 doses) and 46.0%, without recent vaccination (Table 1). Of the 307 contacts with 3-dose vaccination status, 272 had been vaccinated within the previous 3 months.

**Table 1:** Attack rates among household contacts of index patients with SARS-CoV-2 Omicron BA.2 infection in study of vaccine effectiveness against SARS-CoV-2 among household contacts during Omicron BA.2–dominant period, Japan*

Variables	No. household contacts, n = 952	Infected contacts, n = 395	Contact attack rate, %	Risk ratio (95% CI)†
Household contacts				
No. vaccination doses				
0–1	399	198	49.6	Referent
2	246	102	41.5	0.84 (0.70–0.999)
3	307	95	30.9	0.62 (0.51–0.76)
Recent vaccination				
N	637	293	46.0	Referent
Y	315	102	32.4	0.70 (0.59–0.84)
Index patients				
No. vaccination doses				
0–1	515	238	46.2	Referent
2	301	112	37.2	0.81 (0.68–0.96)
3	136	45	33.1	0.72 (0.55–0.93)
Recent vaccination‡				
N	802	343	42.8	Referent
Y	150	52	34.7	0.81 (0.64–1.01)

*Attack rates are stratified by vaccination doses or recent vaccination for household contacts or index patients. †Monovariate analyses. ‡Recent vaccination indicates last vaccine dose <3 months.

The HCAR for contacts of an index patient who had a 2-dose vaccination status was 37.2% and HCAR was 33.1% for contacts of index cases with a 3-dose status. Those results were substantially lower than the 46.2% for contacts of an index patient without complete vaccination status. However, the HCAR for contacts of an index patient with recent vaccination was not substantially lower than that for contacts of an index patient without recent vaccination (Table 1).

Among household contacts who had a 2-dose vaccination status, HCAR was 34.8% for contacts of index patients with complete vaccination but was 49.5% for contacts of index patients without complete vaccination status. However, among household contacts with 3-dose vaccination and without complete vaccination, the HCAR was not much lower for contacts of index patients with complete vaccination than for contacts of index patients without complete vaccination status (Table 2). The HCAR among household contacts without recent vaccination was 37.9% for contacts of index patients with complete vaccination, compared with 52.1% for contacts of index patients without complete vaccination (relative risk 0.73 [95% CI 0.61–0.87]); VE 0.27 [95% CI 0.13–0.39]) (Table 2). We obtained similar results in multivariate analyses (Appendix Table). Among household contacts with recent vaccination, HCAR was not substantially different for contacts of index patients with complete vaccination status and contacts of patients without complete vaccination status (Table 2).

**Table 2:** Household contact attack rates by vaccination status in a study of vaccine effectiveness against SARS-CoV-2 among household contacts during Omicron BA.2–dominant period, Japan*

Vaccination status of household contacts	Vaccination status of index patients	Total no. household contacts	No. infected contacts	Contact attack rate, %	Risk ratio (95% CI)
No. vaccine doses					
0–1	0–1	256	136	53.1	Referent
	2	105	45	42.9	0.81 (0.63–1.03)
	3	38	17	44.7	0.84 (0.58–1.22)
	2–3	143	62	43.4	0.82 (0.66–1.02)
2	0–1	111	55	49.5	Referent
	2	108	39	36.1	0.73 (0.53–0.997)
	3	27	8	29.6	0.60 (0.32–1.10)
	2–3	135	47	34.8	0.70 (0.52–0.95)
3	0–1	148	47	31.8	Referent
	2	88	28	31.8	1.00 (0.68–1.47)
	3	71	20	28.2	0.90 (0.57–1.38)
	2–3	159	48	30.2	0.95 (0.68–1.33)
Vaccination status†					
No recent vaccination	0–1	365	190	52.1	Referent
	2	213	81	38.0	0.73 (0.60–0.89)
	3	59	22	37.3	0.71 (0.51–1.01)
	2–3	272	103	37.9	0.73 (0.61–0.87)
Recent vaccination	0–1	150	48	32.0	Referent
	2	88	31	35.2	1.10 (0.76–1.59)
	3	77	23	29.9	0.93 (0.62–1.41)
	2–3	165	54	32.7	1.02 (0.74–1.41)

*Risk rates are stratified by vaccination doses or recent vaccination for household contacts or index patients. †Recent vaccination indicates last vaccine dose <3 months before illness.

## Conclusions

Our study found the HCAR among household contacts of index SARS-CoV-2 Omicron BA.2 patients with complete vaccination was substantially lower than that among contacts of index patients without complete vaccination. In our previous study on the Omicron BA.1 variant–dominant period, the VE was 0.43 against infectiousness (*5*). Several other previous studies also reported VE against Omicron infectiousness (*10*–*12*); however, the vaccination doses of index patients in those studies might have been associated with the vaccination doses of their household contacts.

Among household contacts without recent vaccination in our study, HCAR was lower for contacts of index patients with complete vaccination status than for contacts of index patients without complete vaccination, a VE of 0.27. However, among household contacts with recent vaccination, we noted no major effect of vaccination completeness for index patients. 

VE was greatly affected by vaccination completeness for index patients among household contacts with 2-dose vaccination but not among household contacts with 3-dose vaccination. Another study in Japan also showed relatively low VE against infectiousness among contacts with 3-dose vaccination compared with VE against infectiousness among other contacts (*13*).

In this study, the HCAR for contacts of an index patient with recent vaccination was not much lower than for contacts of an index patient without recent vaccination. Previous studies have reported relatively stable VE against infectiousness compared with VE against susceptibility (*12*,*14*).

The first limitation of this study is that we did not adjust for confounding factors, such as demographic data, for analyses on all household contacts. The second limitation is that we did not confirm specific variant types with genomic sequencing and different variants might have had different infection rates. Finally, our assessment of risk factors and potential confounders might be limited to the data reported to the PHCs.

In summary, our results indicate that the SARS-CoV-2 Omicron BA.2 attack rate among household contacts without recent vaccination was lower for household contacts of vaccinated index patients than for contacts of unvaccinated index patients. These results strengthen data on indirect effectiveness of COVID-19 vaccines and the value of continued vaccine campaigns to prevent severe illness among index cases and household contacts.

Appendix Additional information on vaccine effectiveness against SARS-CoV-2 among household contacts during Omicron BA.2–dominant period, Japan.
