# Supplementary material for: Vaccine Effectiveness against SARS-CoV-2 among Household Contacts during Omicron BA.2–Dominant Period, Japan
Source: Emerg Infect Dis. 2024 Jul;30(7):1430–3. doi: 10.3201/eid3007.230968 (PMC11210665; doi:10.3201/eid3007.230968)
Supplement: Appendix — Additional information on vaccine effectiveness against SARS-CoV-2 among household contacts during Omicron BA.2–dominant period, Japan. [file 23-0968-Techapp-s1.pdf]

Article DOI: https://doi.org/10.3201/eid3007.230967

EID cannot ensure accessibility for supplementary materials supplied by authors. Readers who have difficulty accessing supplementary content should contact the authors for assistance.

Vaccine Effectiveness against SARS-CoV-2 among Household Contacts during Omicron BA.2–Dominant Period, Japan

Appendix

Appendix Table. Multivariate analyses on attack rates among the household contacts of COVID-19 patients without vaccination within three months*

Variables	Household contacts, n = 637	Infected contacts, n = 293	% Secondary attack rate, median 46.0	Quasi-binomial logistic regression analyses, aOR (95% CI)	Generalized estimating equations, aOR (95% CI)
Risk factors for COVID-19 index patients					
Vaccination					
0–1	365	190	52.1	Referent	Referent
2–3	272	103	37.9	0.64 (0.43–0.95)	0.73 (0.47–1.01)
Sex					
M	290	148	51.0	1.34 (0.96–1.87)	1.31 (0.90–1.90)
F	347	145	41.8	Referent	Referent
Age, y					
≤19	375	182	48.5	1.00 (0.66–1.50)	0.80 (0.50–2.43)
20–59	249	106	42.6	Referent	Referent
≥60	13	5	38.5	0.93 (0.28–3.1)	0.89 (0.23–1.90)
Risk factors of household contacts					
Vaccination					
0–1	389	191	49.1	Referent	Referent

Variables	Household contacts, n = 637	Infected contacts, n = 293	% Secondary attack rate, median 46.0	Quasi-binomial logistic regression analyses, aOR (95% CI)	Generalized estimating equations, aOR (95% CI)
2–3	248	102	41.1	0.75 (0.49–1.13)	0.73 (0.47–1.15)
Sex					
M	313	142	45.4	0.95 (0.69–1.31)	0.92 (0.70–1.20)
F	324	151	46.6	Referent	Referent
Age, y					
≤19	346	162	46.8	0.87 (0.58–1.30)	0.83 (0.57–1.22)
20–59	256	120	46.9	Referent	Referent
≥60	35	11	31.4	0.55 (0.25–1.21)	0.57 (0.24–1.32)
Household size, no. persons					
2	21	9	42.9	0.90 (0.35–2.33)	0.80 (0.31–2.05)
3	71	33	46.5	1.08 (0.63–1.85)	1.09 (0.60–1.99)
≥4	545	251	46.1	Referent	Referent

*All variables were included in the analysis. aOR, adjusted odd ratio.
